# A Short Bowel (Small Intestine = 40 cm), No Ileocecal Valve, and Colonic Inertia Patient Works Well with Oral Intake Alone without Parenteral Nutrition

**DOI:** 10.1155/2014/387307

**Published:** 2014-06-15

**Authors:** Ming-Yi Liu, Hsiu-Chih Tang, Hui-Lan Yang, Sue-Joan Chang

**Affiliations:** ^1^Department of Nutrition, Tainan Sin-Lau Hospital, No. 57, Section 1, Dongmen Road, Tainan City 70142, Taiwan; ^2^Department of Surgery, Tainan Sin-Lau Hospital, No. 57, Section 1, Dongmen Road, Tainan City 70142, Taiwan; ^3^Department of Nursing, Tainan Sin-Lau Hospital, No. 57, Section 1, Dongmen Road, Tainan City 70142, Taiwan; ^4^Department of Life Sciences, College of Bioscience and Biotechnology, National Cheng Kung University, No. 1, University Road, Tainan City 701, Taiwan

## Abstract

We present a 50-year-old male who suffered from ischemic bowel disease, having undergone massive resection of small intestine and ileocecal valve. He had to cope with 40 cm proximal jejunum and 70 cm distal colon remaining. In the postoperative period parenteral nutrition (PN) was used immediately for nutrition support and electrolyte imbalance correction. We gave him home PN as regular recommendation for the short bowel status after discharge from hospital. This patient has tolerated regular oral intake 2 months later and did not develop significant short bowel syndrome. There were several episodes of venous access infection which troubled this patient and admitted him for treatment during home PN. Therefore, we changed home PN to cyclic tapering pattern. The patient could maintain his nutrition and hydration with oral intake alone after tapering home PN 15 months later. He has survived more than one year without PN support and still maintained 80% ideal body weight with average albumin of 3.5 ± 0.2 mg/dL. Although patient was hospitalized every two months to supplement nutrients, however, this has greatly improved the quality of life.

## 1. Introduction

Resection of large portion of the small bowel may cause severe malabsorption, malnutrition, and electrolyte imbalance [1, 2]. The outcomes of the resection depend on the amount of intestine left and the specific section resected and preservation of colonic length or the presence of the ileocecal valve [1]. After immediate postoperative care, keeping the patient alive through the use of parenteral nutrition (PN) and antisecretory agents [3] and promoting gut adaptation by oral nutrition were commonly used [4]. The minimal absorptive area of small intestine to sustain life varies from individual to individual.

## 2. Case Presentation 

**Figure 1:**
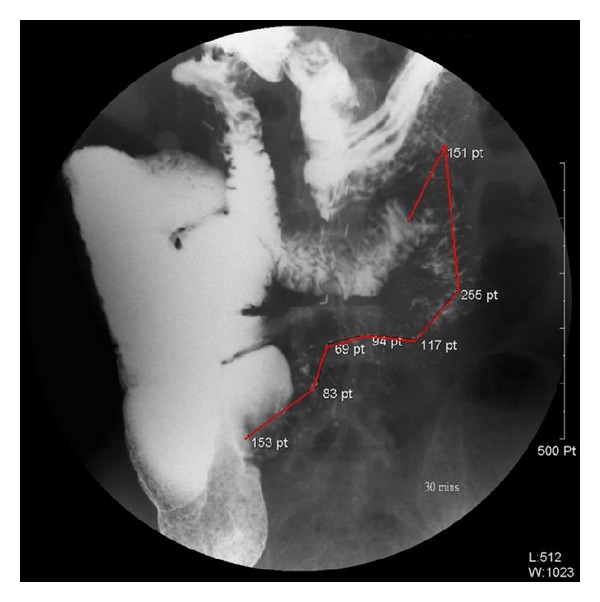
Upper gastrointestinal series show the small intestine about 40 cm (red line).

A 50-year-old homeless male fainted at the station and was sent to the hospital by passerby, height of 153 cm and usual body weight of 54 kg (about three months ago before admission), and complained about abdominal cramping intermitted with poor appetite for several days. The patient's body weight at admission was 40 kg. He suffered from ischemic bowel disease, having undergone massive resection of small intestine and ileocecal valve. He had to cope with 40 cm proximal jejunum (Figure 1) and 70 cm distal colon remaining. Postoperatively his body weight was 34 kg (hospital day 3). He lost weight from 54 kg to 34 kg in 4 months and developed severe malnutrition.

In the postoperative period PN was used immediately for nutrition support and electrolyte imbalance correction. Enteral feeding began 10 days later and antidiarrheal drugs (loperamide 2 mg bid) were used for treatment of diarrhea. Diet progress was clear liquid diet (continue feeding 30 mL/hr for 2 days), diluted elemental diet (continue feeding 700 kcal/day for 3 days), and normal concentration elemental diet (continue feeding 1200 kcal/day for 20 days) to polymeric formulas diet (continue feeding 1200 kcal/day for 5 days). The patient has tolerated oral soft diet and supplement 40 days later. We gave him home PN as regular recommendation and loperamide after being discharged from hospital.

This patient complied with low oxalate and minimizing intraluminal fat diet. These nutrients directly stimulate intestinal adaptation. He has tolerated regular oral intake without antidiarrheal drugs 2 months later. He did not develop significant short bowel syndrome such as increase in bowel movements, malabsorption, anemia, steatorrhea, and muscle wasting. Six months later the patient's body weight was maintained at 42 kg and serum albumin was 3.6 mg/dL. There were several episodes of venous access infection which troubled this patient and admitted him for treatment during home PN. We have evaluated the nutritional status and assured the efficient protein absorption despite the relatively short small intestine. Therefore, we changed home PN to cyclic tapering pattern and monitored body weight, albumin, electrolyte, and hemoglobin level. To our surprise, the patient could maintain his nutrition and hydration with oral intake alone after tapering home PN 15 months later. He has survived more than one year without parenteral support and still maintained 80% ideal body weight (about 41~42 kg) with average albumin of 3.5 ± 0.2 mg/dL. Although the patient weaned the regular PN support, the patient began to develop anemia and vitamin B1 deficiency symptoms. The patient was hospitalized for three days every two months via peripheral parenteral nutrition to supplement vitamins and trace elements.

## 3. Discussion

**Figure 2:**
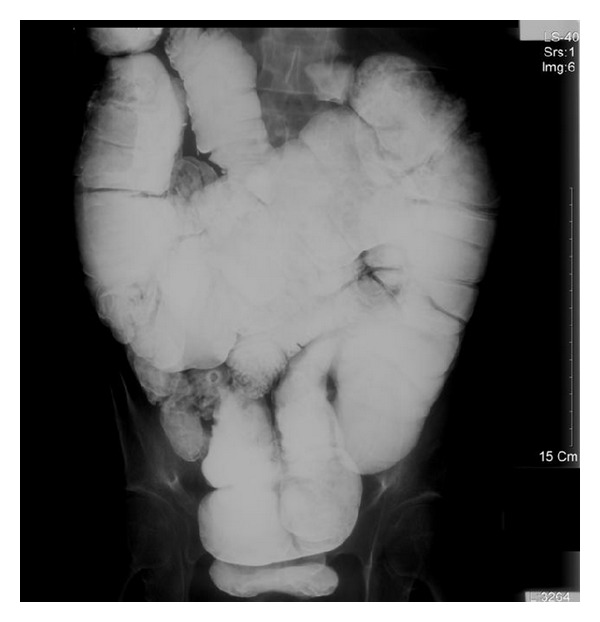
Low gastrointestinal series shows the thick and long colon and the abdomen full of the colon.

Residual intestinal length in these patients weaned from TPN ranged from 27 to 75 cm (mean 57 cm) in pediatric patients [5] and from 57 to 150 cm (mean 96 cm) in adult patients [6]. None of the adult patients with residual small intestinal length less than 40 cm could achieve complete intestinal adaptation. Our patient with 40 cm small intestine and without ileocecal valve was weaned from PN successfully. We further to inspect the intestinal transit time and low gastrointestinal tract (GI tract) series after the patient oral intake alone 16 months. The colon transit times were calculated with radionuclide-filled capsules and an abdominal X-ray. After 64 hours the radionuclides almost did not excrete. The low GI tract series showed that the colon became thick and long. The length from 70 cm became about 120 cm and the abdomen full of the colon (Figure 2). Patient oral intake at liberty but he always complains distension, because nutrition promoting the small intestine and colon adaptation and increased absorptive area. Especially large, thick and inertia colon to prolong the foods stay time that is benefit to absorption.

The major mechanism of luminal proteins is that they are digested into di- and tripeptides for absorption. The oligopeptide transporter PepT1 is responsible for the transport of di- and tripeptides in mammalian intestine [7]. PepT1 is abundant along the small bowel brush border in humans but is expressed at low levels in colonic absorptive cells. Ziegler et al. [8] showed that upregulation of PepT1 in the colon of SBS patients suggests that the human colon can increase the luminal transport of di- and tripeptides during intestinal failure. This patient maintained average albumin of 3.5 ± 0.2 mg/dL. It implied that elevated expression of colonic PepT1 is an example of gut adaptation in this short bowel patient.

## 4. Conclusions

The short bowel and colonic inertia patient receiving enteral and parenteral support for several months increased in the absorptive area and function of the small intestine and colon. This short bowel patient works well with oral intake alone without parenteral nutrition. Although the patient was hospitalized every two months to supplement nutrients, however, this has greatly improved the quality of life.
